# The use of machine learning for investigating the role of plastic surgeons in anatomical injuries: A retrospective observational study

**DOI:** 10.1097/MD.0000000000030943

**Published:** 2022-10-07

**Authors:** Nam Kyu Lim, Jong Hyun Park

**Affiliations:** a Department of Plastic and Reconstructive Surgery, Dankook University College of Medicine, Cheonan, Republic of Korea; b Department of Plastic and Reconstructive Surgery, Dankook University Hospital, Cheonan, Republic of Korea.

**Keywords:** abbreviated injury scale, injury severity score, machine learning, plastic surgery, trauma centers

## Abstract

While plastic surgeons have been historically indispensable in the reconstruction of posttraumatic defects, their role in trauma centers worldwide has not been clearly defined. Therefore, we aimed to investigate the contribution of plastic surgeons in trauma care using machine learning from an anatomic injury viewpoint. We conducted a retrospective study reviewing the data for all trauma patients of our hospital from March 2019 to February 2021. In total, 4809 patients were classified in duplicate according to the 17 trauma-related departments while conducting the initial treatment. We evaluated several covariates, including age, sex, cause of trauma, treatment outcomes, surgical data, and severity indices, such as the Injury Severity Score and Abbreviated Injury Scale (AIS). A random forest algorithm was used to rank the relevance of 17 trauma-related departments in each category for the AIS and outcomes. Additionally, *t* test and chi-square test were performed to compare two groups, which were based on whether the patients had received initial treatment in the trauma bay from the plastic surgery department (PS group) or not (non-PS group), in each AIS category. The department of PS was ranked first in the face and external categories after analyzing the relevance of the 17 trauma-related departments in six categories of AIS, through the random forest algorithm. Of the 1108 patients in the face category of AIS, the PS group was not correlated with all outcomes, except for the rate of discharge to home (*P* < .0001). Upon re-verifying the results using random forest, we found that PS did not affect the outcomes. In the external category in AIS, there were 30 patients in the PS group and 56 patients in the non-PS group, and there was no statistically significant difference between the two groups when comparing the outcomes. PS has contributed considerably to the face and external regions among the six AIS categories; however, there was no correlation between plastic surgical treatment and the outcome of trauma patients. We investigated the plastic surgeons’ role based on anatomical injury, using machine learning for the first time in the field of trauma care.

## 1. Introduction

Trauma is the leading cause of death worldwide, particularly in individuals of reproductive age. These deaths impose a significant economic burden on society, as prime working years are lost in addition to other costs.^[1]^ This underscores the need for organized efforts to address the burden of traumatic events. In South Korea, amendments of laws regarding the “emergency medical service act” in 2012 resulted in the selection of regional trauma centers by the government, with the aim of reducing the preventable trauma death rate (PTDR). These regional trauma centers are modeled upon the trauma centers of nations with advanced healthcare systems, such as the United States or the United Kingdom, and they have now been established in almost every province and with high accessibility.^[2]^

Head injuries are a serious health concern that often result in severe disfigurement, disability, or death.^[3–5]^ Meanwhile, burn injury is also one of the principal causes of death. In the US, such traumatic and burn injuries are the most common cause of death in persons aged 1 to 44 years regardless of socioeconomic background and ethnicity, with hundreds of thousands of cases referred to hospitals annually.^[1,4]^ Notably, the expansion of industry has resulted in an increase in the rate of traumatic accidents in developing nations, such as traffic accidents and gunshot wounds.^[5]^

Plastic surgeons have the skill sets to treat traumatic injuries, including management of soft tissue injuries, fractures in facial structures, neurovascular injuries, and salvaging extremities.^[2,6]^ In 50 years, advancements in diagnostic and therapeutic technologies, such as imaging, microscopy, dressing materials, and surgical instruments, have also been conducive to the role that plastic surgeons play in treating patients with trauma.^[7]^ However, their role in trauma centers globally remains poorly defined despite providing comprehensive care for patients with critical injuries.^[8]^

With the rapid development of machine learning, a more rapid and reliable multi-factor analysis can be performed. A random forest, an ensemble decision method based on random subsets with classification and regression trees, has been verified as a useful model for prediction.^[9]^ Thus, our study aimed to investigate the contribution of plastic surgeons in trauma care using machine learning from an anatomic injury viewpoint.

## 2. Methods

### 2.1. Study participants

This retrospective study adhered to institutional guidelines and was approved by the Institutional Review Board of the Dankook University Hospital (IRB No. 2022-08-016). The study was designed to review data on trauma patients recorded from March 2019 to February 2021 in the Trauma Registry System of our hospital, which has been accredited as a regional trauma center. Of the 5264 patients in the registry, those with missing injury severity score (ISS) values were excluded. Accordingly, 455 patients were excluded. A total of 4809 patients were finally enrolled in the study. The IRB waived all patients’ consent due to the retrospective nature of the study. Participants were classified in duplicate according to the trauma-related departments while conducting the initial treatment. There were seven dedicated departments and ten supportive departments within the trauma-related departments, in accordance with the policies of the trauma care system in South Korea. The detailed information concerning the departments and number of patients in this study are as follows:

1. Dedicated departments (7 departments) included general surgery (GS, 1474 patients), cardiothoracic surgery (CS, 1325 patients), neurosurgery (NS, 1405 patients), orthopedic surgery (OS, 2153 patients), emergency medicine (EM, 949 patients), anesthesiology (AN, 1 patient), and radiology (DR, 40 patients).
2. Supportive departments (10 departments) included plastic surgery (PS, 428 patients), ophthalmology (EY, 117 patients), otorhinolaryngology (ENT, 56 patients), urology (URO, 23 patients), obstetrics and gynecology (OG, 6 patients), internal medicine (IM, 62 patients), pediatrics (PD, 7 patients), psychiatry (PY, 31 patients), rehabilitation medicine (RM, 1 patient), and dental surgery (MS, 150 patients).

### 2.2. Assessments

The trauma registry system of the institution served as a source for information on all trauma patients. Data extracted and used included age, sex, cause of trauma, progression from the emergency room (admission, transfer to another hospital, discharge, and death). The causes of trauma included traffic accidents, falls, slipping, injuries by persons or objects, burns, and unknown causes. In addition, the severity of trauma injury was determined by referring to the Abbreviated Injury Scale (AIS) and Injury Severity Score (ISS), which are well-established anatomical measurements. The AIS assesses six predefined body regions (head and neck, face, thorax, abdomen, extremity, and external), each ranging from 1 (minor) to 6 (maximal). The ISS, another anatomical severity index, was calculated based on the AIS. The ISS ranges from 1 to 75 points.

The assessments of surgical data included the number of patients, number of operations, surgical regions, type of anesthesia, and surgical procedure. Treatment outcomes were evaluated using the duration of hospitalization (length of stay [LOS]: total duration and stay in intensive care unit [ICU]), rate of ICU admission, and post-discharge progress (discharge home, transfer to another hospital, discharge against medical advice, and death).

### 2.3. Random forest analysis

The random forest is composed of many decision trees that were created using a stochastic process. In the classification tree algorithm, a training set of data is divided into nodes to allow a previously unknown record to be appropriately assigned to a class at the end of the procedure. The R program version 4.04 (R foundation, Vienna, Austria) was used to separate the data into two groups: training data (70%) and testing data (30%). We determined that four nodes per tree were needed for optimal correlation of the results. To obtain appropriate stabilization, we used the under-sampling method if one variable contributed to less than 20% of the total sample.

Using this method, we were able to comprehend the relationship between the six AIS categories and outcomes among 17 departments, and it was recognized that the ranking classification was meaningful when the area under curve (AUC) was over 0.7. The strength of the correlation was represented in terms of the variable importance (mean decrease accuracy).

### 2.4. Statistical analysis

In each AIS category, patients were divided into two groups based on whether they had received initial treatment in the trauma bay from the plastic surgery department (PS group) or not (non-PS group). Analysis of variances in sex, cause of trauma, progression from the emergency room, the rate of ICU admission, the number of operations and post-discharge progress was assessed using the chi-square test. Analyses of age, severity score, and duration of hospitalization were conducted using t-test. All statistical significance was set at *P* < .05. Statistical analyses were performed using IBM SPSS Statistics software version 21 (IBM Corp., Armonk, NY).

## 3. Results

### 3.1. Relationship between six categories of AIS and trauma-related departments

**Figure 1.**
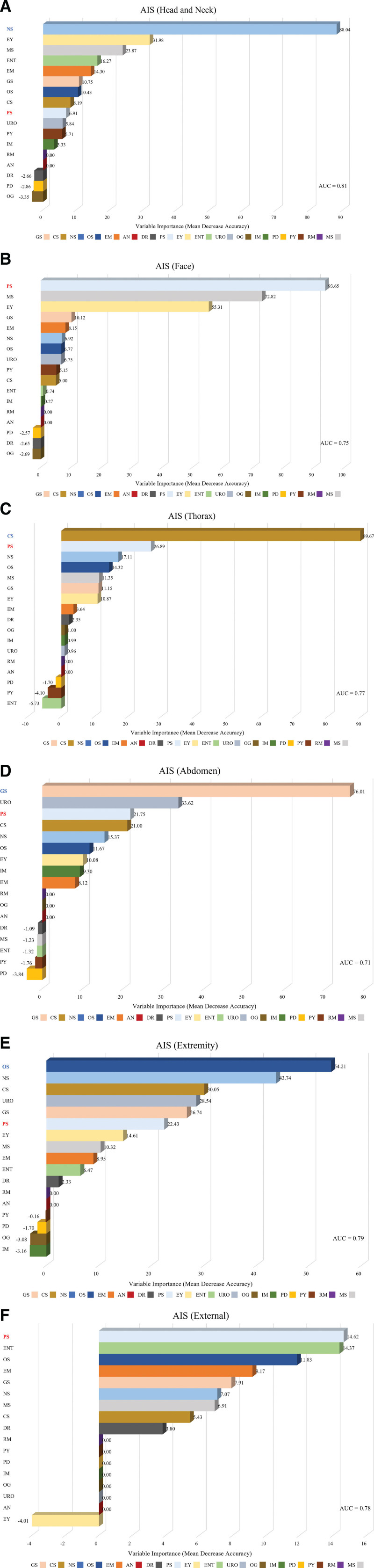
Variable importance among trauma-related departments according to the Abbreviated Injury Scale (AIS) using random forest. (A) Variable importance in head and neck category. (B) Variable importance in face category. (C) Variable importance in thorax category. (D) Variable importance in abdomen category. (E) Variable importance in extremity category. (E) Variable importance in external category. AN = anesthesiology, AUC = area under curve, CS = cardiothoracic surgery, DR = radiology, EM = emergency medicine, ENT = otorhinolaryngology, EY = ophthalmology, GS = general surgery, IM = internal medicine, MS = dental surgery, NS = neurosurgery, OG = obstetrics and gynecology, OS = orthopedic surgery, PD = pediatrics, PS = plastic surgery, PY = psychiatry, RM = rehabilitation medicine, URO = urology.

A total of 4809 patients. The number of patients in each of the six AIS categories was as follows: head and neck (1505 patients, 31.3%), face (1108 patients, 23.0%), thorax (1272 patients, 26.5%), abdomen (884 patients, 18.4%), extremity (3094 patients, 64.3%), and external (86 patients, 1.8%). A random forest algorithm was used to rank the relevance of 17 trauma-related departments in each category of the AIS, as shown in Figure 1. All categories revealed an AUC of 0.7 or higher, indicating the validity of the measurements. The department of plastic surgery (PS) was ranked first in both the face and external categories. Thus, we focused on the face and external regions for the investigation of the plastic surgeons’ role.

### 3.2. PS in the face category of AIS

Of the 1108 patients in the face category of AIS, the plastic surgery (PS group) department treated 364 patients (32.9%) at the initial visit; the 744 patients (67.1%) not treated by the PS department were classified as the non-PS group. There was a predominance of male patients (281 men, 77.2% in the PS group vs 574 men, 77.2% in the non-PS group, *P* = .986) in both groups. The mean age of patients was 51.82 ± 18.96 years (range, 0–92 years) in the PS group and 50.70 ± 20.10 years (range, 0–94 years) in the non-PS group (*P* = .359). Traffic accidents were the most common cause of trauma in both groups, with the PS group having a significantly greater incidence than the non-PS group (213 patients, 58.5% in the PS group, vs 366 patients, 49.2% in the non-PS group, *P* < .0001). According to the anatomical severity analysis, the mean of both the AIS and ISS showed no significant difference between the two groups (AIS, 1.33 ± 0.84 points in the PS group vs 1.38 ± 0.50 points in the non-PS group, *P* = .123; ISS, 13.30 ± 8.81 points in the PS group vs 14.37 ± 11.89 points in the non-PS group, *P* = .093).

After initial treatment in a trauma bay, over 95% patients were admitted to our hospital (356 patients, 97.8% in the PS group vs 711 patients, 95.6% in the non-PS group, *P* = .006), and there were 20 patients that died in the non-PS group. During hospitalization, there was no statistically significantly difference between the two groups in terms of total LOS, ICU LOS, and the rate of ICU admission (total LOS, 19.30 ± 21.39 days in the PS group vs 17.90 ± 19.73 days in the non-PS group, *P* = .289; ICU LOS, 5.68 ± 9.67 days in the PS group vs 7.29 ± 11.53 days in the non-PS group, *P* = .082; the rate of ICU admission, 52.0% in the PS group vs 48.3% in the non-PS group, *P* = .862) (Table 1).

**Table 1:** Characteristics of trauma patients classified as having Abbreviated Injury Scale (AIS) in the face category according to the plastic surgery (PS) treatment.

Variable	Total (n = 1108)		
	PS (n = 364)	Non-PS (n = 744)	*P* value
Age (mean ± SD, yr)	51.84 ± 18.96	50.70 ± 20.10	<.359*
Sex			
Male	281 (77.2%)	574 (77.2%)	<.986†
Female	83 (22.8%)	170 (22.8%)	
Cause of trauma			
Traffic accident	213 (58.5%)	366 (49.2%)	<.0001†
Falls	62 (17.0%)	130 (17.5%)	
Slipping	24 (6.6%)	94 (12.6%)	
Injuries by persons or objects	61 (16.8%)	134 (18.0%)	
Burn	2 (0.5%)	0 (0.0%)	
Unknown	2 (0.5%)	20 (2.7%)	
AIS (mean ± SD, points)	1.33 ± 0.84	1.38 ± 0.50	<.123*
ISS (mean ± SD, points)	13.30 ± 8.81	14.37 ± 11.89	<.093*
Progress in emergency room			
Admission	356 (97.8%)	711 (95.6%)	<.006†
Transfer	8 (2.2%)	13 (1.7%)	
Discharge	0 (0.0%)	0 (0.0%)	
Death	0 (0.0%)	20 (2.7%)	
Total LOS (d)	19.30 ± 21.39	17.90 ± 19.73	<.289*
ICU admission (n)	185(52.0%)	374(48.3%)	<.862†
ICU LOS (d)	5.68 ± 9.67	7.29 ± 11.53	<.082*
Operation			
Number of patients	166 (45.6%)	69 (9.3%)	<.0001†
Number of surgical procedures	259	103	
Post-discharge progress			
Home	238 (65.4%)	406 (54.6%)	<.0001†
Transfer	110 (30.2%)	243 (32.7%)	
AMA	2 (0.5%)	7 (0.9%)	
Death	6 (1.6%)	52 (7.0%)	
Etc.‡	8 (2.2%)	36 (4.8%)	

AIS = Abbreviated Injury Scale, ICU = intensive care unit, ISS = Injury Severity Score, LOS = length of stay, n = number of patients, SD = standard deviation.

* *t* test.

† Chi-square test.

‡ It included unrecorded (due to no admission in our hospital) and unidentified cases.

**Figure 2.**
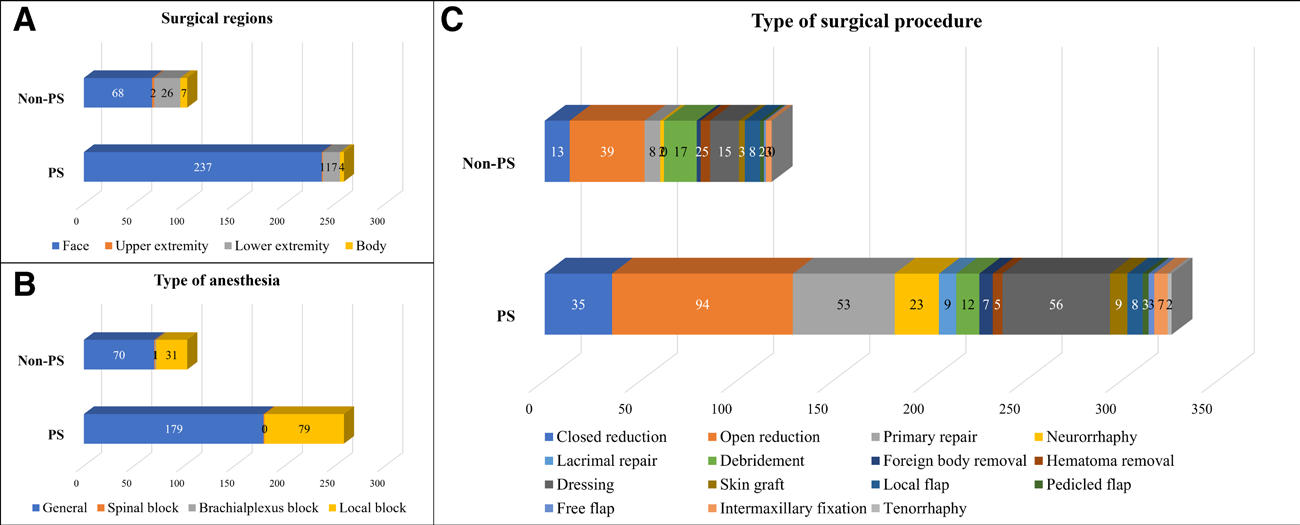
Surgical data analysis in the face category according to the plastic surgery (PS) treatment. (A) Surgical regions. (B) Type of anesthesia. (C) Type of surgical procedure.

There was a significant difference between the two groups regarding the number of patients that underwent plastic surgical treatment (166 patients, 45.6% in the PS group vs 69 patients, 9.3% in the non-PS group, *P* < .0001). To elaborate, 259 cases in the PS group and 103 cases in the non-PS underwent plastic surgical treatment, with the average surgical procedure per operation being 1.26 ± 0.51 in the PS group and 1.15 ± 0.35 in the non-PS group respectively, with a statistically significant difference between the groups (*P* = .017). The face was the area most frequently operated on in both groups (237 cases, 91.5% in the PS group vs 68 cases, 66.0% in the non-PS group, *P *< .0001), and general anesthesia was the preferred anesthetic method in both groups (179 cases, 69.1% in the PS group vs 70 cases, 68.0% in the non-PS group, *P* = .393). In both groups, open reduction was the most commonly performed surgical treatment in terms of type of procedure, followed by dressing in the PS group and debridement in the non-PS group, among all procedures (Fig. 2).

Upon completion of hospitalization treatment, the rate of discharge to home was the highest in both groups, with a statistically significant difference between groups (238 patients, 65.4% in the PS group vs 406 patients, 54.6% in the non-PS group, *P* < .0001) (Table 1).

### 3.3. PS in the external category of AIS

Of the 86 patients in the external category of AIS, the plastic surgery (PS group) department treated 30 patients (34.9%) at the initial visit; the 56 patients (65.1%) not treated by the PS were classified as the non-PS group. There was a predominance of male patients (22 men, 73.3% in the PS group vs 40 men, 71.4% in the non-PS group, *P* = .851) in both groups. The mean age of patients was 46.03 ± 19.37 years (range, 13–83 years) in the PS group and 55.21 ± 19.52 years (range, 8–86 years) in the non-PS group (*P* = .040). Regarding causes of trauma, traffic accidents were the most common cause in the non-PS group while burns were the most common cause in the PS group (*P* = .028). According to the anatomical severity analysis, the mean value for both AIS and ISS showed no significant difference between the two groups (AIS, 1.87 ± 1.22 points in the PS group vs 1.64 ± 1.31 points in the non-PS group, *P* = .443; ISS, 8.90 ± 13.83 points in the PS group vs 12.98 ± 14.03 points in the non-PS group, *P* = .200).

After initial treatment in an emergency room, over 76% patients were admitted to our hospital (23 patients, 76.7% in the PS group vs 44 patients, 78.6% in the non-PS group, *P* = .338), and there were 3 deaths recorded in the non-PS group. During hospitalization, there was no statistically significant difference between the two groups in terms of total LOS, ICU LOS, and the rate of ICU admission (total LOS, 20.26 ± 21.23 days in the PS group vs 22.72 ± 22.26 days in the non-PS group, *P* = .665; ICU LOS, 2.58 ± 4.03 days in the PS group vs 7.05 ± 10.05 days in the non-PS group, *P* = .089; the rate of ICU admission, 40.0% in the PS group vs 17.9% in the non-PS group, *P* = .695) (Table 2).

**Table 2:** Characteristics of trauma patients classified as having Abbreviated Injury Scale (AIS) in the external category according to the plastic surgery (PS) treatment.

Variable	Total (n = 86)		
	PS (n = 30)	Non-PS (n = 56)	*P* value
Age (mean ± SD, yr)	46.03 ± 19.37	55.21 ± 19.52	<.040*
Sex			
Male	22 (73.3%)	40 (71.4%)	<.851†
Female	8 (26.7%)	16 (28.6%)	
Cause of trauma			
Traffic accident	5 (16.7%)	22 (39.3%)	<.028†
Falls	0 (0.0%)	4 (7.1%)	
Slipping	0 (0.0%)	4 (7.1%)	
Injuries by persons or objects	2 (6.7%)	5 (8.9%)	
Burn	12 (40.0%)	13 (23.2%)	
Unknown	11 (36.7%)	8 (14.3%)	
AIS (mean ± SD, points)	1.87 ± 1.22	1.64 ± 1.31	<.443*
ISS (mean ± SD, points)	8.90 ± 13.83	12.98 ± 14.03	<.200*
Progress in emergency room			
Admission	23 (76.7%)	44 (78.6%)	<.338†
Transfer	7 (23.3%)	9 (16.1%)	
Discharge	0 (0.0%)	0 (0.0%)	
Death	0 (0.0%)	3 (5.4%)	
Total LOS (d)	20.26 ± 21.23	22.72 ± 22.26	<.665*
ICU admission (n)	12 (40.0%)	10 (17.9%)	<.695†
ICU LOS (d)	2.58 ± 4.03	7.05 ± 10.05	<.089*
Operation			
Number of patients	16 (53.3%)	10 (17.9%)	<.0001†
Number of surgical procedures	55	47	
Post-discharge progress			
Home	14 (46.7%)	19 (33.9%)	<.443†
Transfer	6 (20.0%)	20 (35.7%)	
AMA	1 (3.3%)	1 (1.8%)	
Death	2 (6.7%)	3 (5.4%)	
Etc.‡	7 (23.3%)	13 (23.2%)	

AIS = Abbreviated Injury Scale, ICU = intensive care unit, ISS = Injury Severity Score, LOS = length of stay, n = number of patients, SD = standard deviation.

* *t* test.

† Chi-square test.

‡ It included unrecorded (due to no admission in our hospital) and unidentified cases.

**Figure 3.**
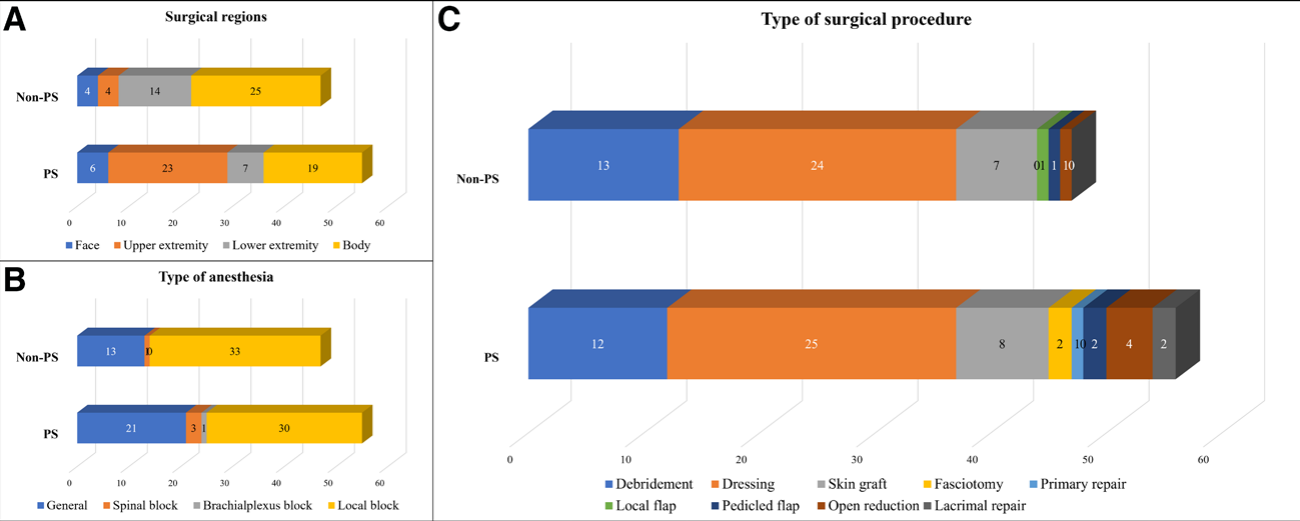
Surgical data analysis in the external category according to the plastic surgery (PS) treatment. (A) Surgical regions. (B) Type of anesthesia. (C) Type of surgical procedure.

There was a significant difference between the two groups in the number of patients who underwent plastic surgical treatment (16 patients, 53.3% in the PS group vs 10 patients, 17.9% in the non-PS group, *P* = .0001). That is, 55 cases in the PS group and 47 cases in the non-PS underwent plastic surgical procedures, with the average surgical procedure per operation being 1.02 ± 0.14 in the PS group and 1.00 ± 0.00 in the non-PS group (*P* = .358). The number of cases that underwent procedures in the sum of the upper extremity and body regions was significantly higher in the PS group than in the non-PS group (44 cases, 76.3% in the PS group vs 29 cases, 63.7% in the non-PS group, *P* = .001). The most common type of anesthesia was local anesthesia in both groups (30 cases, 54.5% in the PS group vs 33 cases, 70.2% in the non-PS group, *P* = .331). In both groups, dressing was the most commonly performed surgical treatment regarding type of procedure, follow by debridement and skin graft, among all procedures (Fig. 3).

Upon completion of treatment, the PS group showed a higher rate of discharge to home (14 patients, 46.7% in the PS group vs 19 patients, 33.9% in the non-PS group), whereas the non-PS group had a higher proportion of transfer to another hospital (6 patients, 20.0% in the PS group vs 20 patients, 35.7% in the non-PS group). Despite this, there was no significant difference between the two groups (*P* = .443) (Table 2).

### 3.4. Relationship between post-discharge progress and trauma-related departments in the face category of AIS

**Figure 4.**
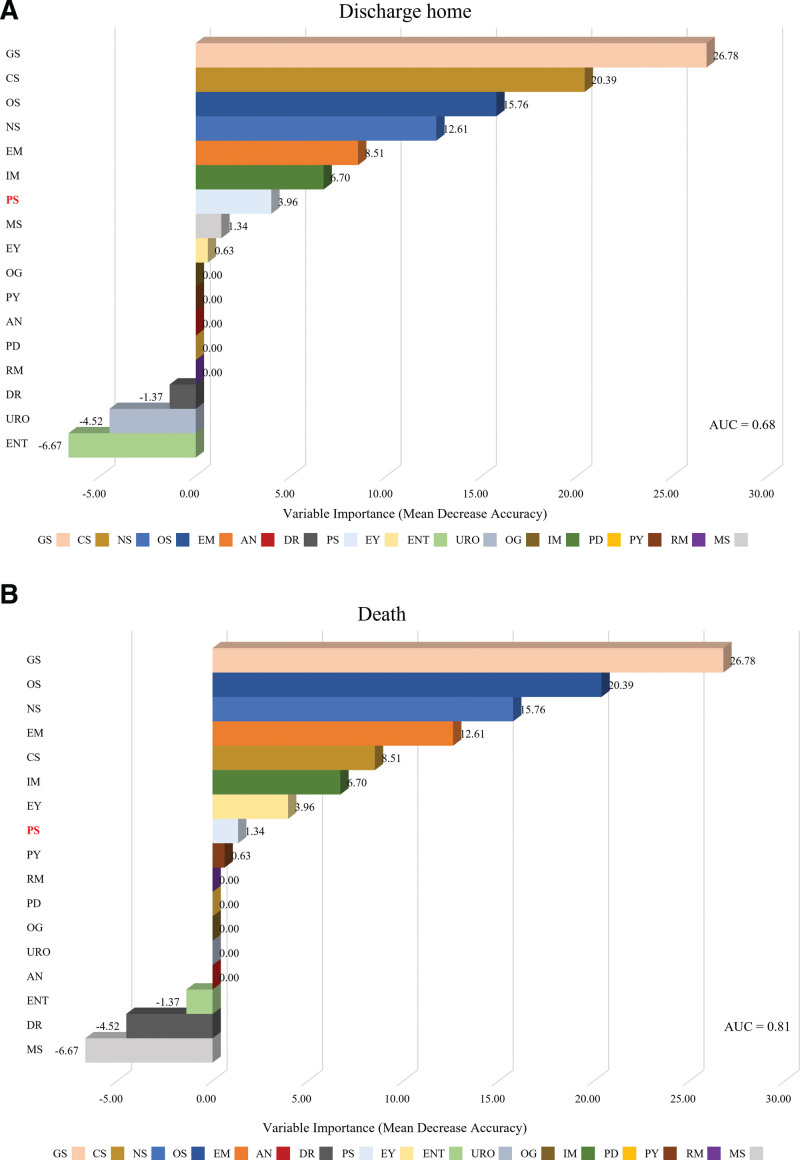
Variable importance among trauma-related departments according to the post-discharge progress in face category of Abbreviated Injury Scale (AIS) using random forest. (A) Variable importance in discharge home. (B) Variable importance in death. AN = anesthesiology, AUC = area under curve, CS = cardiothoracic surgery, DR = radiology, EM = emergency medicine, ENT = otorhinolaryngology, EY = ophthalmology, GS = general surgery, IM = internal medicine, MS = dental surgery, NS = neurosurgery, OG = obstetrics and gynecology, OS = orthopedic surgery, PD = pediatrics, PS = plastic surgery, PY = psychiatry, RM = rehabilitation medicine, URO = urology.

Plastic surgery contributed considerably to the face and external regions among the six AIS categories. In both categories, plastic surgeons had a high rate of participation in the initial treatment, which led to a high rate of operation. However, the overall high rate of operation in plastic surgery did not correlate with the outcomes, except for the PS group’s high rate of discharge to home for the face category of AIS. Therefore, we used random forest analysis to verify the hypothesis that there was a correlation between operation and discharge to home after plastic surgical treatment. However, undergoing plastic surgery had no correlation with discharge to home. Rather, five dedicated departments (GS, CS, NS, OS, and EM) were predominantly associated with the discharge to home. However, the AUC was <0.7; hence, the reliability of the result was low. Meanwhile, these departments also had a strong correlation with death, with an AUC value of 0.81 (Fig. 4).

## 4. Discussion

International armed conflicts such as the First and Second World Wars and the Korean and Vietnamese wars have also catalyzed developments in motion trauma systems. Prompt evacuation, early resuscitation, and referral to competent surgical centers constitute critical lessons of these global conflicts. The American College of Surgeons acknowledged the need for an organized response to trauma and established the Committee on Treatment on Fractures in 1922, subsequently named the Committee on Trauma. Several papers discussing preventable deaths with more timely and adequate care promoted the establishment of a more structured and methodical system to manage severe trauma.^[10]^ The initial guidance on trauma care proposed by the American College of Surgeons in 1976 served as the foundation for current Level I trauma centers.^[2,4,10]^ Subsequent years to early 2000s saw the establishment of “trauma systems” in every state in the US.^[10]^

Multiple definitive care facilities in the trauma care system form a network that covers the full range of care for all injuries, ordered into four levels of trauma centers in the US. Level I trauma facilities provide tertiary care and are central to the trauma care system. All patients who require resources either have direct access or are efficiently transported via a streamlined transfer process to reach Level I care. The facilities offer end-to-end total care, from prevention to rehabilitation, and must have sufficient material and human resources to fulfill their central roles.^[11,12]^ Level I facilities are equipped to treat patients with the most challenging traumatic injuries and are, therefore, resourced with a full range of specialist surgeons, including orthopedic, neurovascular, microvascular, cardiac, thoracic, plastic, hand, obstetric and gynecologic surgeons, as well as ophthalmologists, otolaryngologists, and urologists. Complex craniofacial injuries require multidisciplinary treatment, whereas maxillofacial trauma cases are treated by otorhinolaryngologists, oral maxillofacial surgeons, plastic surgeons, and ophthalmologists according to the Advanced Trauma Life Support (ATLS) protocol.^[3,4,12]^

In South Korea, a 35% PTDR was reported in 2010, which is more than twice the rate of PTDR in other advanced economies at that time. Thus, the amendments of laws regarding the “emergency medical service act” in 2012 resulted in the selection of regional trauma centers by the government, with the aim of reducing PTDR. Since 2014, between one and five regional trauma centers have been established annually, and as a result, a total of 15 facilities were operative in 2020 (17 centers have been selected until 2020). All centers are supervised by the Department of Trauma Management at the National Emergency Medical Center.^[13]^ Organizing trauma teams that include “activation teams” is essential to ensure efficient and effective operations in regional trauma centers. The activation team, which includes at least two trauma surgeons, should be in the trauma bay within 10 minutes after the arrival of a patient with severe trauma. “Activation” of the trauma team is determined by the physiological and anatomical criteria and mechanisms of trauma as presented in Table 3. In response to these efforts, the PTDR in South Korea reduced rapidly in the 2010s (30.5% in 2015 to 19.9% in 2017).

**Table 3:** The activation criterion of regional trauma center.

1.Physiological criteria A.Respiratory obstruction/Hypopnea/Airway intubation state B.Adult: respiratory rate < 10 or > 30 times/min or systolic blood pressure < 90 mm Hg or Heart rate > 100 bpm C.Glasgow coma scale < 13 D.Need blood transfusion
2.Anatomical criteria A.Penetration: Head and neck, chest, abdomen, extremity(proximal to elbow and knee) B.Flail chest C.Open or depressed skull fracture D.Paralysis E.Orthopedics: Pelvic bone fracture, Two or more proximal long-bone fractures, Crushed, degloved, or mangled extremity, Amputation proximal to wrist and ankle
3.Trauma mechanism A.Fall: Adult > 20 feets, Children > 10 feets B.High-risk auto crash i.Intrusion; >12 inches, occupant site; >18 inches any site ii.Ejection (partial or complete) from automobile iii.Death in same passenger compartment iv.Vehicle telemetry data consistent with high risk of injury C.Traffic accident: pedestrian/Bicyclist thrown, run over, or with significant impact (>20 mph)
4.Emergency medical service provider judgement

The government funds the nation’s trauma center, which includes funding for full-time dedicated specialists and on-call compensation. At present, only seven departments are eligible for the position of dedicated specialists: GS, CS, NS, OS, EM, DR, and AN.^[2,13]^ Simultaneously, the regional trauma center maintains a roster of on-call specialists from EY, ENT, URO, OG, IM, PD, PY, RM, MS, and PS. These specialists act as supporting specialists whose employment in the trauma center is not financed by the government.^[13]^ Meanwhile, the current disparity in government financing for multidisciplinary approaches continues to be criticized. Indeed, the influential contribution of plastic surgeons in trauma care is underappreciated as a result of regulations prohibiting them from serving as dedicated specialists, depriving them of adequate compensation.

Patients with maxillofacial injuries are frequently treated at trauma centers worldwide. According to a 1995 analysis by the Major Trauma Outcome Study, 34% of trauma patients suffered mid-face injuries, while 25% sustained facial bone fractures.^[14]^ Facial injuries range in severity from minor chin lacerations and chipped teeth to life-threatening injuries involving the entire panfacial area that require complex airway management and extended intensive care, including surgery.^[3,15,16]^ In children, facial fractures may cause permanent damage. Indeed, many pediatric maxillofacial patients suffer from malformations and functional disabilities. In the US, facial injuries cost $1.2 billion in healthcare expenses annually.^[14,17]^ The complexity of craniofacial injuries necessitates specialist care by plastic surgeons, as evidenced by the fact that roughly 40% of such injuries are treated by plastic surgeons, compared to 5% by general surgeons.^[5]^

In addition to the expertise provided by plastic surgeons for patients with complex facial injuries, plastic surgeons play a pivotal role in soft tissue reconstruction.^[18–20]^ A report on Level I trauma centers in India indicated that plastic surgeons treated various anatomical areas, including the upper limb (49%), lower limb (35%), head and neck (8%), and trunk (8%).^[21]^ Further, another study demonstrated a wide range of reconstructions in plastic surgery, including significant injuries in the extremities (40%), general wound care (36.4%), and craniofacial surgery (16.4%).^[8]^ According to Fox et al, procedures performed by plastic surgeons include delayed primary suturing, tangential excision of necrotic tissue, skin graft or flap coverage, and release of secondary scar contractures to manage soft tissue injuries.^[22]^ In our study, we found a strong relationship between the face and external injuries and plastic surgery, with the open reduction in the face area and the dressing in the external area being the most frequently performed procedures.

However, defining a certain department’s role in the assessment of trauma patients affected by many factors is challenging. In this study, we applied machine learning to overcome the limitations of conventional statistical methods of analysis. Today, with the rapid development of the data processing, various machine learning techniques were introduced, allowing a more accurate and reliable multi-factor analysis. Among them, a random forest, an ensemble decision method based on random subsets with classification and regression trees has been verified as useful model for prediction.^[9]^ All of the data can be used for training and validation while avoiding decision-tree overfitting, and when data are absent, the random-forest approach is relatively powerful when multicollinearity occurs.^[23]^ A tree that has no correlation with another has three types of nodes: the root, the internal, and the leaf. Each tree is built from all data creating the root node, and subsequent splits determined the order of importance of the predictor variables. Data are randomly separated into two groups, namely training dataset and test dataset. The training dataset is utilized to construct the random forest model using three parameters, including the number of trees created, the number of predictor variables used in each tree, and the size of each node. Then the data dataset is applied in order for this model to be validated. The test dataset can be used to make predictions, with the final prediction result being the average of all predicted values from multiple classification trees. As a result, mean decrease accuracy is calculated to estimate the relevant importance of variables within the random forest model.^[23,24]^ Simultaneously, the reliability of the prediction correlation is evaluated to the area under curve (AUC), and in general, if it is 0.7 or more, it is regarded as a reliable result.^[23]^

To our knowledge, this study was the first application of the random forest algorithm to investigate the role of trauma patients. We not only found that plastic surgery was highly related to the facial and external areas, but also deduced that there was no correlation between the plastic surgical procedure and the rate of discharge to home in facial trauma patients. The latter result, in particular, was achieved by a combined evaluation of conventional statistical analysis and machine learning. Nevertheless, this study has several limitations. First, due to the retrospective nature of this study, it is not representative of South Korean regional trauma centers in general. Second, the severity of trauma patients could be evaluated in various viewpoints, but this study focused on anatomical criteria only. In general, the severity of trauma injuries is assessed according to anatomical, physiological, and comorbidity systems. The ISS and AIS are used as indicators of anatomical severity, while the Glasgow Coma Scale (GCS) and Revised Trauma Score (RTS) are physiological indices. The trauma and injury severity score (TRISS) and severity characterization of trauma (ASCOT) reflect both anatomical and physiological properties.^[25,26]^ Among them, we analyzed the trauma severity in patients using anatomical indicators such as AIS and ISS. Third, we excluded other variables that could have influenced patients’ outcomes, such as past medical history and family history, because obtaining such personal information from trauma patients at the initial evaluation was difficult. Lastly, depending on the hospital, the triage of clinical tasks may vary. At our hospital, for example, hand trauma is triaged by the PS and OS, while mandibular fractures are triaged by the PS and MS.

We investigated the plastic surgeons’ role according to anatomical injury using machine learning. Plastic surgery had contributed considerably to the face and external regions among the six AIS categories; however, there was no correlation between plastic surgical treatment and outcome of trauma patients. Nevertheless, we found that the random forest algorithm is a useful method for multivariate analysis and that when it is used in combination with conventional statistical analysis methods, it supports the interpretation of the results. To our knowledge, this study was the first application of random forest algorithm in investigating the role of plastic surgeons in the field of trauma care.

## Acknowledgments

I would like to express my appreciation to Professor Jae Woo Chung (Department of Neurosurgery, Dankook University College of Medicine) for contributing to the in-depth discussion about machine learning.

## Author contributions

**Conceptualization:** Nam Kyu Lim.

**Data curation:** Nam Kyu Lim, Jong Hyun Park.

**Formal analysis:** Nam Kyu Lim.

**Funding acquisition:** Nam Kyu Lim.

**Investigation:** Nam Kyu Lim.

**Methodology:** Nam Kyu Lim.

**Project administration:** Nam Kyu Lim.

**Resources:** Nam Kyu Lim.

**Software:** Nam Kyu Lim.

**Supervision:** Nam Kyu Lim.

**Validation:** Nam Kyu Lim.

**Visualization:** Nam Kyu Lim.

**Writing – original draft:** Nam Kyu Lim.

**Writing – review & editing:** Nam Kyu Lim.

## References

[1] Surrusco M Tong W Roden KS. The impact of an independent transfer center on the evaluation and transport of patients with burn and maxillofacial injuries to definitive care at a level 1 trauma center. Ann Plast Surg. 2012;68:484–8. 22531403 10.1097/SAP.0b013e31823b69c2

[2] Jung JS Kang DH Lim NK. Epidemiology of severe trauma patients treated by plastic surgeons: a 7-year study at a single regional trauma center in South Korea. Arch Plast Surg. 2020;47:223–7. 32453930 10.5999/aps.2020.00430 PMC7264906

[3] Bagheri SC Dimassi M Shahriari A. Facial trauma coverage among level I trauma centers of the United States. J Oral Maxillofac Surg. 2008;66:963–7. 18423287 10.1016/j.joms.2008.01.020

[4] Drolet BC Tandon VJ Ha AY. Unnecessary emergency transfers for evaluation by a plastic surgeon: a burden to patients and the health care system. Plast Reconstr Surg. 2016;137:1927–33. 27219245 10.1097/PRS.0000000000002147

[5] Currie KB Ross P Collister P. Analysis of scalp and forehead injuries in a level I trauma center. J Craniofac Surg. 2017;28:1350–3. 28538063 10.1097/SCS.0000000000003585

[6] Chung S Zimmerman A Gaviria A. Plastic surgery response in natural disasters. J Craniofac Surg. 2015;26:1036–41. 26080117 10.1097/SCS.0000000000001658

[7] Luce EA Hollier LH Lin SJ. Plastic surgeons and the management of trauma: from the JFK assassination to the Boston Marathon bombing. Plast Reconstr Surg. 2013;132:1330–9. 24165614 10.1097/PRS.0b013e3182a7094c

[8] Peterson SL Moore EE. The integral role of the plastic surgeon at a level I trauma center. Plast Reconstr Surg. 2003;112:1371–5; discussion 1377, discussion 78. 14504523 10.1097/01.PRS.0000082815.79881.51

[9] Liu Y Zhao H. Variable importance-weighted Random Forests. Quant Biol. 2017;5:338–51. 30034909 PMC6051549

[10] David JS Bouzat P Raux M. Evolution and organization of trauma systems. Anaesth Crit Care Pain Med. 2019;38:161–7. 29476943 10.1016/j.accpm.2018.01.006

[11] Gittings DJ Mendenhall SD Levin LS. A decade of progress toward establishing regional hand trauma centers in the United States. Hand Clin. 2019;35:103–8. 30928043 10.1016/j.hcl.2018.12.001

[12] Committee on Trauma American College of Surgeons. Resources for Optimal Care of the Injured Patient. Chicago, IL: American College of Surgeons, 2014.

[13] Lim NK Kang DH. Plan for plastic surgeons to participate in trauma teams at regional trauma and emergency centers. J Korean Med Assoc. 2018;61:710–4.

[14] The Korean Society of Traumatology. Chapter 18. Face, Textbook of Trauma. 1st edition. Seoul, South Korea: Panmuneducation, 2018:251–84.

[15] Ricci JA Vargas CR Ho OA. The impact of major league baseball on the incidence of operative hand and facial trauma at a level 1 trauma center. Arch Plast Surg. 2019;46:198–203. 30940793 10.5999/aps.2018.00276 PMC6536876

[16] Lee H Kim KS Choi JH. Trauma severity and mandibular fracture patterns in a regional trauma center. Arch Craniofac Surg. 2020;21:294–300. 33143397 10.7181/acfs.2020.00556 PMC7644353

[17] Gebran SG Wasicek PJ Elegbede A. Characterization of age-related injury patterns and surgical treatment of pediatric facial fractures: analysis of the national trauma data bank. J Craniofac Surg. 2019;30:2189–93. 31369513 10.1097/SCS.0000000000005789

[18] Hendrickson SA Khan MA Verjee LS. Plastic surgical operative workload in major trauma patients following establishment of the major trauma network in England: a retrospective cohort study. J Plast Reconstr Aesthet Surg. 2016;69:881–7. 27025358 10.1016/j.bjps.2016.02.003

[19] Thakar HJ Pepe PE Rohrich RJ. The role of the plastic surgeon in disaster relief. Plast Reconstr Surg. 2009;124:975–81. 19730322 10.1097/PRS.0b013e3181b17a7a

[20] Arifi HM Duci SB Zatriqi VK. A retrospective study of 572 patients with hand burns treated at the Department of Plastic Surgery Kosovo during the period 2000-2010. Int J Burns Trauma. 2014;4:7–13. 24624309 PMC3945823

[21] Singhal M Naalla R Dave A. The role of plastic and reconstructive surgeon in trauma care: perspectives from a Level I trauma centre in India. Indian J Plast Surg. 2018;51:170–6. 30505087 10.4103/ijps.IJPS_212_17 PMC6219353

[22] Fox JP Markov NP Markov AM. Plastic surgery at war: a scoping review of current conflicts. Mil Med. 2021;30:e327–35. 10.1093/milmed/usaa361 33206965

[23] Alderden J Pepper GA Wilson A. Predicting pressure injury in critical care patients: a machine-learning model. Am J Crit Care. 2018;27:461–8. 30385537 10.4037/ajcc2018525 PMC6247790

[24] Wang J Shi L. Prediction of medical expenditures of diagnosed diabetics and the assessment of its related factors using a random forest model, MEPS 2000-2015. Int J Quan Health Care. 2020;32:99–112. 10.1093/intqhc/mzz135 32159759

[25] Jung K Lee CJ Kim J. Injury severity scoring system for trauma patients and trauma outcomes research in Korea. J Acute Care Surg. 2016;6:11–7.

[26] The Korean Society of Traumatology. Chapter 4. Injury Severity Scoring, Textbook of Trauma. 1st ed. Seoul, South Korea: Panmuneducation, 2018:27–34.

